# Donor and Recipient Age-Mismatches: The Potential of Transferring Senescence

**DOI:** 10.3389/fimmu.2021.671479

**Published:** 2021-04-28

**Authors:** Jasper Iske, Tomohisa Matsunaga, Hao Zhou, Stefan G. Tullius

**Affiliations:** ^1^ Division of Transplant Surgery & Transplant Surgery Research Laboratory, Brigham and Women´s Hospital, Harvard Medical School, Boston, MA, United States; ^2^ Department of Urology, Osaka Medical College, Osaka, Japan

**Keywords:** immunosenescence and inflammaging, immunosenescence, immune aging, senescent cell, passenger leukocytes, senescent associated secretory phenotype

## Abstract

In transplantation, donor and recipients frequently differ in age. Senescent cells accumulate in donor organs with aging and have the potential to promote senescence in adjacent cells when transferred into recipient animals. Characteristically, senescent cells secrete a myriad of pro-inflammatory, soluble molecules as part of their distinct secretory phenotype that have been shown to drive senescence and age-related co-morbidities. Preliminary own data show that the transplantation of old organs limits the physical reserve of recipient animals. Here, we review how organ age may affect transplant recipients and discuss the potential of accelerated aging.

## Introduction

Organ transplantation is the treatment of choice for end-stage-organ failure. The supply of organs, however, is limited, resulting in prolonged waiting times with many patients dying or becoming too ill to be transplanted. Aging demographics have incrementally affected the deceased donor population with older donors showing the by far largest proportional increase. Organs from older donors are, at the same time, underutilized, frequently discarded or not even considered (1).

The most obvious strategy that may close the gap between demand and supply may thus be an optimized utilization of older organs from deceased donors (2, 3). Increased donor age, at the same time poses a significant risk for adverse outcomes including more frequent rejections due to an augmented immunogenicity in aging (4, 5). Most relevantly, older organs have shown compromised long-term graft outcomes with inferior graft survival rates in addition to increased rates of chronic allograft dysfunction in kidney, heart and lung transplantation (6).

Senescent cells accumulate with aging and have been identified as critical in driving the immunogenicity of older organs linked to the accumulation of cell-free mitochondrial-DNA that accelerate alloimmune responses (7). Recent evidence also suggests that senescent cells can induce a senescent phenotype in adjacent cells, a potential mechanism on how the engraftment of older organs may facilitate the spread of senescence. Depletion of senescent cells, at the same time, has been shown to ameliorate a wide range of age‐associated disabilities and diseases (8–12).

Here, we introduce potential mechanisms and consequences of prompting bystander senescence and discuss clinically relevant aspects of senescent cell spread when transplanting older organs. Although speculative, age-disparate transplantation may also provide unique opportunities as the transplantation of young organs may contribute to rejuvenation.

## Senescent Cells Accelerate Aging

Cellular senescence is characterized as a stable and terminal state of growth arrest based on acquired anti-apoptotic pathways (SCAPS, termed senescent cell anti-apoptotic pathways) that render senescent cells resistant to apoptosis (13). Thus, senescent cells accumulate in many tissues with aging (14–16). Notably, stem cells critical for tissue regeneration have also been shown to undergo senescence associated with the loss of their self-renewal capacity considered as a driver of age-related tissue dysfunction and organismal aging (17–19). Characteristics of senescent cells include distinct transcriptional signatures with upregulated genes causing cell cycle arrest, epi-genetic modifications and a divers non-coding RNA profile (20). Senescence cells also have a compromised mitochondrial function (21) and an altered lysosomal activity with overexpression of endogenous lysosomal beta-galactosidase, serving as the most widely used biomarker to visualize senescent cells, termed senescence-associated β-galactosidase (Sa-β-gal) (22). Of note, high expression of Sa-β-gal is not always associated with cellular senescence as it has also been detected in cells undergoing quiescence (23, 24), which – in contrast to senescence – displays a reversible cell-cycle arrest required for tissue repair and regeneration.

Senescent cells exhibit a distinct, pro-inflammatory secretome consisting of cytokines (IL-6, IL-8, TNF-α), chemokines (CCL2, CCL20) and matrix remodeling enzymes termed the “Senescent Associated Secretory Phenotype” (SASP) (25). The production of SASP is a cardinal feature of senescent cells contributing to age‐related tissue dysfunction, chronic age-associated diseases and organismal aging, impairing tissue homeostasis and impeding neighboring cell function (26).

Cellular senescence can be triggered by oncogenic and DNA‐damaging stressors that induce DNA damage responses, a signaling pathway in which ATM or ATR kinases block cell-cycle progression through stabilization of p53 and transcriptional activation of the cyclin-dependent kinase inhibitors p21 (27). Moreover, the cyclin-dependent kinase inhibitor p16^INK4a^ facilitates cell cycle arrest and can therefore also be used as a senescent cell marker (28). As a consequence of DNA damage, senescent cells exhibit increased frequencies of DNA damage foci containing phosphorylated histone H2A.X that are preferentially located at the telomeres and thus termed telomere associated foci (TAF) (29).

Strikingly, senescent cells are capable of auto-inducing a senescent phenotype in surrounding, non-senescent, bystander cells *via* gap junction mediated cell–cell contact and processes involving reactive oxygen species (ROS). Thus, continuous exposure of intact fibroblasts to senescent cells resulted in increased numbers of DNA double-strand breaks (DSBs) indicating senescence, which had been inhibited when blocking gap junction-mediated cell–cell contact (30).

SASP appears to play a critical role in driving bystander senescence. Quantitative proteomics with small molecule screens in transwell two chamber experiments that co-cultured naive human fibroblasts with senescent fibroblasts identified various components of the SASP including TGF-β family ligands, VEGF, CCL2 and CCL20, all capable of inducing paracrine senescence (31). Moreover, culturing naive fibroblasts with conditioned medium derived from senescent fibroblasts demonstrated comparable effects. The senescent phenotype remained detectable 14 days after splitting both cell lines indicating long-term effects (31). A broad range of additional SASP components including IGFBP-7, PAI-1, IL-6 and CXCR2-binding chemokines (such as IL-8 or GROα) have also been shown to drive senescence (32–35).

The spread of senescence has also been confirmed *in vivo* utilizing transgenic Sos Egfr^wa2/+^ mice that develop papillomas with a senescent phenotype within their basal and suprabasal layers. Although there were no senescent cells in the tissue close to normal skin, increased frequencies of senescent cells had been detected in surrounding tissue adjacent to senescent papillomas (31).

## Can the Engraftment of Old Organs Promote Senescence?

We have previously shown that older donor organs bear increased frequencies of senescent cells (7). Thus, when transplanting an older organ, an increased number of senescent cells is transferred to recipients posing the potential to accelerate senescence. In support of this hypothesis, intraperitoneal transplantation of relatively small numbers of senescent cells into young mice resulted into an augmentation of senescence in visceral adipose tissue associated with a compromised physical capacity (36). In detail, senescent cells from luciferase expressing transgenic mice were intraperitoneally injected and assessed by quantifying SA-βgal^+^, p16Ink4a^+^ and TAF^+^ cells in visceral adipose tissue. By two months, amounts of SA-βgal^+^ and p16Ink4a^+^ cells but also luciferase negative TAF^+^ cells had increased, indicating an augmented number of senescent cells of recipient origin. Consistent with the spread of senescence, distant tissues including the quadriceps muscles displayed an increased frequency of the senescent cell markers such as p16Ink4a, TNF-α, and IL-6 (36). Moreover, autologous transplantation of senescent cells into healthy knee joints promoted the development of an osteoarthritis‐like condition in young mice (37). These observations are consistent with our own preliminary data showing a compromised physical capacity in young mice that had received an old cardiac isograft. Furthermore, when transferring senescent cells into the skeletal muscle of immunocompromised NOD SCID gamma mice, increased numbers of senescent cells and augmented SASP-marker expression including IL‐1α, IL‐1β, IL‐6 and TNF‐α had been detected (38).

**Figure 1:**
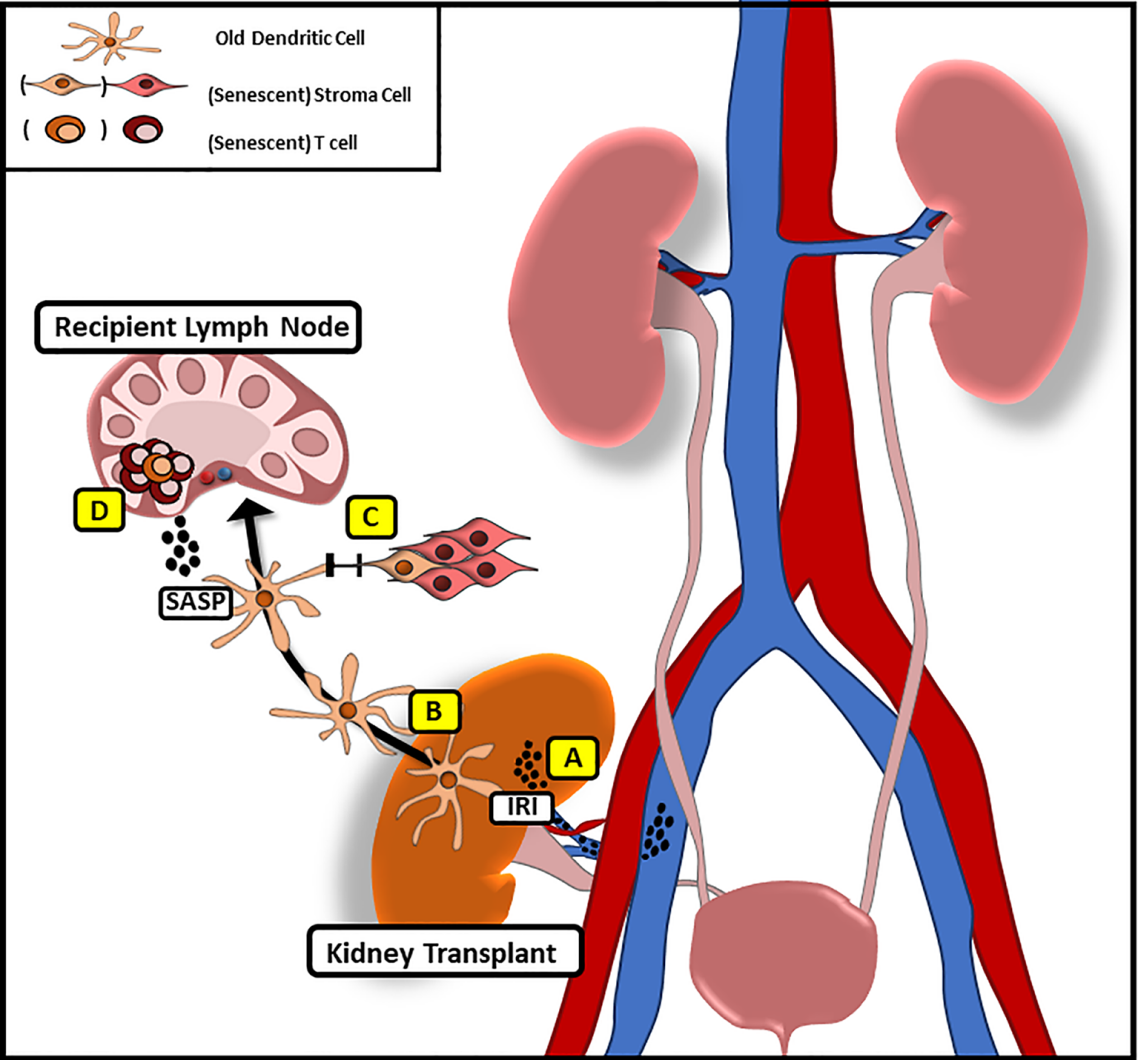
Potential Mechanism of Transferring Senescence Following Solid Organ Transplantation. **(A)** Following IRI pro-inflammatory factors with similarities to SASP are released that may promote systemic senescence in the recipient. **(B)** Donor derived, old dendritic cells migrate to recipient lymph nodes following implantation to initiate alloimmune responses through direct antigen presentation. **(C)** Via gap junction mediated cell–cell contact old DC may promote senescence in recipient stroma cells **(D)** while inducing a senescent phenotype in recipient T cells through the release of SASP-factors.

Following organ transplantation, significant numbers of passenger leukocytes deriving from the transplanted organ have been shown to disseminate into the recipient tissue (39–42), supporting the concept that senescence may be transferred in organ transplantation (**Figure 1**).

## Ischemia Reperfusion Injury as a Driver of SASP Promoting Senescence

Ischemia reperfusion injury (IRI) displays an inevitable feature of organ transplantation promoting a sterile inflammation linked to the release of various pro-inflammatory cytokines coinciding with the production of SASP by senescent cells. It appears thus possible that IRI may aid to the promotion of senescence in transplant recipients.

The rapid increase in oxygen demand within the ischemic tissue subsequent to organ reperfusion induces oxidative stress, mitochondrial damage and electrolyte imbalance associated with local inflammation including the release of ROS (43), pro-inflammatory cytokines, in particular TNF-α, IL-1, IL-6 and IL-8 (44, 45) in addition to various proteases (46). Notably, IL-1α expression has been shown to induce an inflammasome mediated SASP activation with the secretion of IL-6 and IL-8 that reinforce senescent growth arrest (31, 47). It has also been demonstrated that ROS induce senescence by promoting mitochondrial dysfunction directly through damaging mitochondrial DNA. Alternatively, ROS may also facilitate senescence in synergy with modifications of the telomerase reverse transcriptase enzyme in addition to the activation of p53 and Ras pathways (48). ROS also inhibit autophagy *via* p53 activation and the induction of micro-RNAs, effects that further amplify mitochondrial dysfunction (49, 50). Reconciling these cellular effects of IRI on senescence induction, a recent study has confirmed that IRI induces senescence in both cardiomyocytes and interstitial cell populations of murine hearts both within the infarct and in the peri-infarct region of the left ventricular myocardium (51).

## Can Senolytic Drugs Inhibit the Spread of Senescence?

Senolytics are a class of drugs that selectively clear senescent cells through inhibiting their SCAPs, thus driving them into apoptosis. According to their targeted SCAP, first generation senolytics can be subdivided in groups including, i), BCL-2 inhibitors such as navitoclax that inhibit the pro-survival pathway BCL-2/BCL-xL, ii), PI3AK/AKT inhibitors including Dasitinib and Quercetin (D & Q) that constrain the synergistic effects of PI3K and Akt inactivating the apoptosis mediating proteins Bad, caspase-9 and FKHRL1 and iii), FOXO regulators such as Foxo4-DRI interfering with the inhibition of p53-mediated apoptosis (52). In addition, cardiac glycosides have been shown to exert senolytics activity through targeting the Na^+^/K^+^ Atpase (53, 54) while most recently a BET family protein degrader targeting the non-homologous end joining and autophagy has been delineated as a promising novel senolytic drug (55) (**Table 1**).

**Table 1:** Reported senolytic drugs.

Senolytic Drug	Target	Reference
**Dasitinib/Quercetin**	**PI3K/AKT pathway**	Xu et al. (36)
**Fisetin**		Yousefzadeh et al. (56)
**Luteolin/Curcumin**		Yousefzadeh et al. (56)
**17-DMAG**	**HSP90-PI3K/AKT pathway**	Fuhrmann-Stroissnigg et al. (57)
**Navitoclax**	** **	Zhu et al. (58)
**A1331852/A1155463**	**BCL family**	Zhu et al. (59)
**Panobinostat**	** **	Samaraweera et al. (60)
**FOXO4-DRI**	**P53/FOXO4 interaction**	Baar et al. (12)
**Catechins**	**Bax/Bcl-2, Nrf2, PI3K/AKT/mTOR pathways**	Kumar et al. (61)
**Cardiac Glycosides (Ouabain, Proscillaridin A, Digoxin)**	**BCL Family and Na^+^/K^+^ ATPase**	Triana-Martínez et al. (54) Guerrero et al. (53)
**BETd**	**NHEJ/autophagy**	Wakita et al. (55)

PI3K, Phosphoinositide 3-kinase; AKT, Protein Kinase B; 17-DMAG, 17-Dimethylaminoethylamino-17-demethoxygeldanamycin; HSP90, Heat Shock Protein 90’ FOXO4-DRI, Forkhead box protein O4 peptide D-Retro Inverso Isoform; Bax, Bcl-2-associated X protein; Nrf2, Nuclear factor erythroid 2-related factor 2; mTOR, mechanistic Target of Rapamycin; Na^+^/K^+^ ATPase, sodium–potassium adenosine triphosphatase; BETd, bromodomain and extra-terminal domain family protein degrader; NHEJ, non-homologous end joining.

Experimentally, senolytics have been shown to ameliorate a broad range of age-associated pathologies including diabetes (62), cardiovascular disease (11, 63), Alzheimer disease (64), osteoporosis (8) and cancer (55). Moreover, clinical studies focusing on idiopathic fibrosis, complications of advanced diabetes, osteoarthritis and Alzheimer have been initiated (65). Of note, senolytic drugs such as navitoclax that only target a single SCAP are more likely to exert substantial off-target apoptotic effects on non-senescent cell types including platelets and immune cells while eliminating only a restricted range of senescent cells (58). Thus, efforts have been made to develop novel senolytic drugs that act on multiple SCAPs, increasing the specificity for senescent cells while reducing off-target effects.

Routes of application may play an additional role: site specific delivery of quercetin improved pancreas islet transplant outcome with enhanced glycemic control by delaying cellular senescence in a mouse model of diabetes (66). Moreover, combinatorial treatment with D & Q has been shown to deplete senescent cells in old murine donor organs, thus decreasing ischemia reperfusion derived release of SASP factors which translated into dampened allo-immune responses, prolonging transplant survival (7). The clearance of senescent cells in donor organs may not only exert anti-inflammatory effects but also restrain a potential transfer of senescence underscoring the therapeutic potential of these drugs in organ transplantation. As a proof of concept, administration of D & Q to mice that had received intraperitoneal injections of senescent cells prevented the spread of senescence and resulting physical dysfunction (36). Senolytic treatment of transplant donors may decrease the pool of senescent cells while a subsequent treatment of the recipient may ensure sustainable clearance of senescent cells, preventing a spread of senescence following transplantation. In addition, senomorphic drugs such as ATM kinase inhibitors (67) and Janus kinase inhibitors (68) that attenuate SASP may, in turn, restrain senescence induction through systemic SASP factors released upon ischemia reperfusion injury when transplanting older organs (69). Of relevance, not all senescent cells may promote age-related complications as recent reports have delineated a physiological hemostatic role of a distinct senescent endothelial cell population in liver sinusoids (70).

## Envisioning Rejuvenation When Transplanting Young Organs Into Old Recipients

In contrast to the potential of transferring senescence to recipients, it may also be possible that a young donor organ may exert rejuvenating effects when transplanted into an old recipient. We wish to point out that this concept is theoretical at this point, nevertheless worthwhile to speculate on.

Experimental data have shown that young parabiotic animals have the capacity to rejuvenate brain, heart and muscle function of old mice (71–73). Moreover, transferring plasma derived from young into old mice increased neuronal plasticity of the hippocampus and improved cognitive functions providing support for the concept of rejuvenation (74). However, subsequent studies aiming to delineate soluble factors within the blood that may mediate rejuvenation have not yielded clear results (75–77).

Tissue derived factors may be particularly relevant for exerting rejuvenation. Extracellular vesicles (EVs), for instance, containing RNAs, proteins, and lipid components that may be released by transplanted tissue stem cells, have been shown to promote stem cell plasticity and tissue regeneration (78–82). Notably, EVs derived from young mesenchymal stroma cells (MSCs) rejuvenated old endothelial progenitor cells (EPCs) *in vitro*, while EVs derived from old MSCs lacked this capacity. Furthermore, miRNA-126-loaded EVs have been able to rejuvenate senescent EPCs *in vitro* (83).

At least in theory, adult stem cells that have been detected in multiple recipient tissues including muscle, liver, myocardium and endothelium following hematopoietic stem cell transplantation (84–86) may support organ function through transdifferentiating into dysfunctional recipient tissue. However, there is no evidence at this time that donor derived MSCs integrated into recipient tissue following solid organ transplantation.

## Conclusion

The transplantation of older donor organs is associated with more frequent acute rejection rates and compromised outcomes calling for age-specific treatment approaches that may improve the quality of older organs while reducing immunogenicity. At the same time transplanting an organ from an old donor may pose a risk for young recipients. The clinical relevance and significance of this concept appears obvious and demands therefore a thorough evaluation. The potential of rejuvenation when transplanting young donor organs, in turn, are of theoretical consideration and will also demand a detailed analysis. Critical will therefore be studies that probe either accelerated aging or rejuvenation in relevant pre-clinical transplant models utilizing immunosuppression.

Optimizing the use of available organs for transplant including those from older donors continues to be of critical importance to close the gap between demand and supply in organ transplantation. Utilizing senolytic drugs that selectively deplete senescent cells may constitute a potential approach to improve the outcomes of older organs while restricting the spread of senescence.

## Author Contributions

All authors listed have made a substantial, direct, and intellectual contribution to the work and approved it for publication.

## Conflict of Interest

The authors declare that the research was conducted in the absence of any commercial or financial relationships that could be construed as a potential conflict of interest.
